# Instruments to Assess Preferences for Everyday Living of Older Adults With Various Care Needs: An Evidence Map

**DOI:** 10.1093/geroni/igab046.1017

**Published:** 2021-12-17

**Authors:** Mike Rommerskirch-Manietta, Daniel Purwins, Kimberly Van Haitsma, Katherine Abbott, Martina Roes

**Affiliations:** 1 DZNE, German Center for Neurodegenerative Diseases, Nordrhein-Westfalen, Germany; 2 DZNE, Witten, Nordrhein-Westfalen, Germany; 3 The Pennsylvania State University, University Park, Pennsylvania, United States; 4 Miami University, Oxford, Ohio, United States; 5 German Center for Neurodegenerative Diseases (DZNE), Witten, Nordrhein-Westfalen, Germany

## Abstract

Background: Instruments to identify and assess preferences for everyday living are important tools for health professionals. For research purposes, they appear equally essential, for example, to develop new care approaches based on the preferences of the older adults. So far, it seemed unknown which instruments already exist to identify and assess preferences for everyday living. Method: We conducted an evidence map to identify instruments, to understand how preferences are assessed and which instruments are focusing everyday living. Results: We plotted our results in the form of a bubble plot. We identified instruments that map multiple domains (e. g. function and leisure activities) or only one topic (e. g. food, personal hygiene or brightness). Preferences are assessed using direct questions, frequencies, sorting, stimuli, or even scores. Our results show the variety of how preferences are defined and the range of instruments to assess preferences for everyday living of older adults.

